# CAREGIVERS WITH MODIFIABLE RISK FACTORS FOR DEMENTIA: LEVERAGING EXISTING PUBLIC HEALTH STRATEGIES

**DOI:** 10.1093/geroni/igad104.0460

**Published:** 2023-12-21

**Authors:** Janelle Gore, John Omura, Roshni Patel, Benjamin Olivari, Lisa McGuire

**Affiliations:** Centers for Disease Control and Prevention, Atlanta, Georgia, United States; Centers for Disease Control and Prevention, Atlanta, Georgia, United States; Cyberdata Technologies (CDC Contractor), Atlanta, Georgia, United States; CDC, Atlanta, Georgia, United States; CDC, Atlanta, Georgia, United States

## Abstract

Caregivers are more likely to self-report subjective cognitive decline, an early indicator of possible future Alzheimer’s disease and related dementias (ADRD), than non-caregivers. Research has identified potential modifiable risk factors for ADRD. To estimate the prevalence of eight modifiable risk factors for ADRD (i.e., high blood pressure, physical inactivity, obesity, type 2 diabetes, depression, current smoking, hearing loss, and binge drinking) among caregivers and non-caregivers, this study analyzed 2021 BRFSS data from 40 jurisdictions that conducted the caregiver module. Among respondents aged ≥18 years (n=232,772), we estimated prevalence of risk factors among caregivers and non-caregivers by selected demographic and health characteristics and the distribution of these characteristics by caregiving status and health condition of care recipient. Significant differences were assessed using t-tests (p< 0.05). Overall, caregivers reported significantly higher prevalence of six out of eight modifiable risk factors for ADRD examined (i.e., high blood pressure, obesity, diabetes, depression, current cigarette smoking, hearing loss) compared with non-caregivers, including high blood pressure (38.8% versus 32.6%), depression (25.1% versus 18.4%), and cigarette smoking (17.2% versus 12.1%). Caregivers of persons living with ADRD reported high blood pressure, diabetes, depression, and hearing loss more often than other types of caregivers. These findings suggest caregivers, especially caregivers of persons living with ADRD, can benefit from existing state-based public health strategies to reduce chronic conditions, such as heart health education and programming. The management of chronic diseases is also beneficial to the brain health of caregivers.

